# Enhanced *Synechococcus* export associated with diatom sinking

**DOI:** 10.1093/ismeco/ycag183

**Published:** 2026-07-14

**Authors:** Takehiro Shimonaka, Taketoshi Kodama, Shigeyoshi Otosaka, Taku Wagawa, Takafumi Kataoka, Junya Hirai, Kazutaka Takahashi

**Affiliations:** Graduate School of Agricultural and Life Sciences, The University of Tokyo, Tokyo 113-8657, Japan; Graduate School of Agricultural and Life Sciences, The University of Tokyo, Tokyo 113-8657, Japan; Atmosphere and Ocean Research Institute, The University of Tokyo, Kashiwa 277-8654, Japan; Fisheries Resources Institute, Japan Fisheries Research and Education Agency, Niigata 951-8121, Japan; Faculty of Marine Science and Technology, Fukui Prefectural University, Obama 917-0003, Japan; Atmosphere and Ocean Research Institute, The University of Tokyo, Kashiwa 277-8654, Japan; Graduate School of Agricultural and Life Sciences, The University of Tokyo, Tokyo 113-8657, Japan

**Keywords:** biological carbon pump, *Synechococcus*, diatom, aggregates, fecal pellets

## Abstract

Phytoplankton sinking represents a major component of oceanic biological carbon sequestration, commonly referred to as the biological carbon pump (BCP). Despite their small size and lack of mineral ballast, pico-sized cyanobacteria are transported to the deep ocean and contribute to carbon sequestration. However, the mechanisms governing their sinking remain poorly understood. To address this knowledge gap, we investigated sinking eukaryotic and cyanobacterial communities and their DNA-based fluxes in zooplankton fecal pellets and non-fecal particles using metabarcoding and qPCR analyses targeting the 16S and 18S rRNA genes. Cyanobacteria, mainly *Synechococcus* subcluster 5.1 clade I, accounted for 3.11%–46.2% of phytoplankton reads in sinking particles, with their contribution and flux peaking during the spring diatom bloom. Diatoms were the largest contributors among phytoplankton and a dominant component of the eukaryotic assemblages in sinking particles. A substantial proportion of the sinking cyanobacterial flux was observed in the non-fecal particle fraction. The cyanobacterial sinking flux was not associated with satellite-derived surface cyanobacterial biomass but was significantly and positively correlated with the diatom sinking flux. These results suggest that the incorporation of cyanobacteria into diatom-containing aggregates represents a major pathway by which they contribute to the BCP, especially during the spring bloom. Our findings demonstrate positive feedback of diatom biomass on the BCP and highlight that modulation of interactions among phytoplankton groups regulates cyanobacterial sinking.

## Introduction

A fraction of phytoplankton-fixed CO_2_ is transported to the deep ocean through biological processes, thereby contributing to long-term carbon sequestration [1]. These processes collectively constitute the biological carbon pump (BCP), which is primarily driven by the gravitational sinking of particulate organic carbon (POC) [2]. A percentage of the total surface primary production is exported below the mixed layer [3], while most of the POC is remineralized in the upper mesopelagic zone [4]. A fraction of the POC transported below the mesopelagic zone is effectively sequestered from the atmosphere for centuries or longer [2]. Therefore, the proportion of POC transported to the mesopelagic zone is a key factor determining the efficiency of carbon sequestration by the BCP.

Pico-sized cyanobacteria, represented by *Synechococcus* and *Prochlorococcus*, are estimated to account for approximately one quarter of oceanic primary production and constitute the second-largest contributors after diatoms [5, 6]. Traditionally, these organisms were thought not to directly contribute to the BCP because of their small size and lack of mineral ballast [7]. However, a modeling study suggested that picoplankton sinking contributes substantially to the BCP [8]. This view has been supported by observations detecting phycoerythrin fluorescence and cyanobacterial genetic markers in sinking particles [9–11]. Furthermore, recent work has revealed that the sinking efficiency of these cyanobacteria varies with season and location [12]. Given their substantial contribution to primary production, understanding the factors controlling their sinking flux is essential for clarifying the variables affecting the efficiency of carbon sequestration by the BCP. Quantitative contributions remain less well understood; however, proposed processes driving cyanobacterial sinking include (i) zooplankton grazing, (ii) cyanobacterial aggregation, and (iii) aggregation with other plankton [13–17]. Thus, classification of sinking particles is essential for understanding the mechanisms controlling cyanobacterial sinking flux.

Combining morphological identification with molecular approaches provides new insights into biological interactions within sinking particles [14, 18, 19]. Previous studies [20–24] have classified sinking particles into non-fecal particles (mainly composed of phytodetritus and zooplankton remains) and fecal pellets, which can be further categorized by shape reflecting their producers. For example, cylindrical pellets are produced by large copepods or krill [20, 25], and ellipsoidal pellets are produced by appendicularians [26]. This approach enabled the identification of particle sinking pathways to the deep ocean [20–24]. However, organisms present within these particles are difficult to identify morphologically due to the loss of diagnostic features resulting from degradation and fragmentation [9]. In contrast, molecular analyses, particularly metabarcoding, enable a comprehensive assessment of community structure, even in sinking particles affected by degradation and predation [9]. Metabarcoding analyses lack quantitative evaluation; therefore, combining them with quantitative PCR (qPCR) can overcome this limitation by providing absolute or relative abundance estimates.

In this study, we aimed to identify the taxonomic groups that play key roles in cyanobacterial sinking. To achieve this objective, we collected sinking particles at a depth of 890 m over a 1-year period in the mid-latitude western Pacific marginal sea (the Sea of Japan), where strong seasonal variability leads to pronounced changes in the biomass and community composition of phytoplankton and zooplankton [27, 28]. We then classified particles by morphology and conducted metabarcoding and qPCR analyses for each particle type.

## Materials and methods

### Sampling and environmental data collection

**Figure 1:**
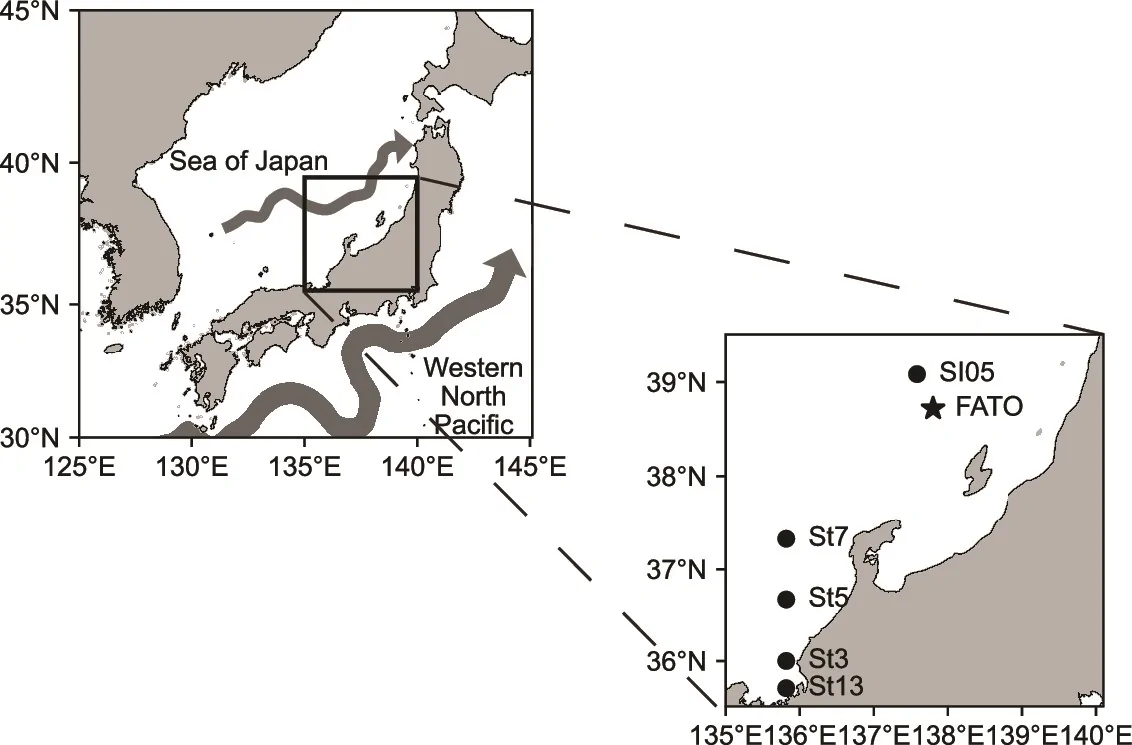
Map of the sampling stations. The star on the small-scale map (right) denotes the sediment trap station (FATO). The closed circles denote the offshore stations in Wakasa Bay (St3, St7, St9, and St13) and Stn. SI05, where suspended particles from the euphotic zone were collected. Arrows in the large-scale map denote the major ocean surface currents in the region: the Kuroshio Current in the western North Pacific and the Tsushima Warm Current in the Sea of Japan.

Carousel-type sediment traps (NiGK SMD-6000W), equipped with a 50-cm-diameter conical collector (0.19625 m^2^) with a 2-cm mesh, were moored at a depth of 890 m at Stn. FATO (38° 43.2′ N, 137° 48′ E; seafloor depth: 1777 m) in the southern Sea of Japan (Fig. 1), corresponding to the sampling location used by Shimonaka et al. [14]. Sinking particles were collected by sequentially switching 13 polypropylene receiving cups (310 ml; I-boy, AS ONE) filled with 5% neutral formalin at 26-day intervals from 22 June 2022 to 25 May 2023. Chemical analyses of mass flux, POC flux, particulate organic nitrogen (PON) flux, and the stable isotope ratios of carbon and nitrogen were conducted as described by Shimonaka et al. [14] after removal of swimmers.

Vertical profiles of temperature and salinity at Stn. FATO during the sampling period were obtained from the MOVE/MRI.COM-JPN Dataset (10.20783/DIAS.639) [29], and an 8-day composite net primary production (NPP) was derived from CAFE (Carbon, Absorption, and Fluorescence Euphotic-resolving) model calculations [30] based on MODIS-Aqua satellite data (http://www.science.oregonstate.edu/ocean.productivity/). Surface chlorophyll *a* concentrations for each phytoplankton taxonomic group at Stn. FATO were obtained from ocean-color satellite data (10.48670/moi-00280). Phytoplankton biomass estimated from satellite data is restricted to the surface layer and does not represent the entire water column. However, in the Sea of Japan, the vertical composition of phytoplankton remains relatively consistent throughout the year [27]. Therefore, surface-layer chlorophyll *a* concentration is considered to reflect seasonal variations in the biomass of phytoplankton groups throughout the water column. In addition, to investigate the seasonal succession of the phytoplankton community in the Sea of Japan, we retrieved 16S rRNA gene amplicon sequencing data of suspended particles collected from the euphotic zone throughout the year at offshore stations in Wakasa Bay (accession number PRJDB20594; Fig. 1), as well as data collected in June 2022 at Stn. SI05, located near Stn. FATO (accession number PRJDB18587; Fig. 1), from the DDBJ BioProject database.

### Fecal pellets subsampling

The volume and number of fecal pellets in sinking particles were determined using a stereo microscope (MZ16, Leica) and a digital microscope (VHX-8000, Keyence). A 0.25–1 ml stirred solution was collected from each receiving cup, and fecal pellets longer than 100 μm were visually classified into three shape-based categories: cylindrical pellets considered to originate from large copepods or krill [20, 25], ellipsoidal pellets considered to originate from appendicularians [26], and others (Figure S1). The “others” category included pellets with alternative morphologies (e.g. spherical), as well as pellets whose shapes could not be clearly identified due to degradation or fragmentation. The length and width of each fecal pellet were measured, and pellet volume was calculated from these dimensions following Li et al. [22]. Fecal pellet volume was subsequently converted to carbon content following Shimonaka et al. [14].

### Amplicon sequencing and qPCR analysis

To determine the taxonomic composition of sinking particles and their sinking flux, amplicon sequencing and qPCR analyses were conducted. Five types of subsamples were prepared: (i) cylindrical pellets, (ii) ellipsoidal pellets, (iii) other pellets, (iv) non-fecal particles, and (v) bulk samples. For each fecal pellet category, three to five pellets were pooled to generate a single subsample per receiving cup. Non-fecal particles consisted of the remaining sinking particles after all fecal pellets were removed from a 0.2 ml aliquot of the sample, whereas bulk samples comprised particles collected from a separate 0.2 ml aliquot. Triplicate subsamples were prepared for each sample type. The carbon content of fecal pellet samples was calculated following the method described in subsection Fecal Pellets Subsampling and that of non-fecal particles and bulk samples was calculated based on the sample splitting ratio. Subsamples were rinsed with DNase/RNase-free water (Qiagen) to remove formalin, preserved in 99.5% ethanol, and DNA was extracted using a QIAamp DNA FFPE Tissue Kit (Qiagen) following the manufacturer’s protocol.

Amplicon sequencing targeting the primer pair 515Y (5′-GTGYCAGCMGCCGCGGTAA-3′) and 926R (5′-CCGYCAATTYMTTTRAGTTT-3′) [31], which amplifies both 16S (targeting prokaryotes and chloroplasts) and 18S rRNA genes (targeting eukaryotes) [32], was performed following the protocol described by Shimonaka et al. [14]. At that time, other pellets were excluded from the analysis because they were mixtures of degraded ellipsoidal or cylindrical fecal pellets and pellets of other shapes, indicating that they did not originate from a single source and contributed little to the POC flux. The first PCR reaction was performed using a polymerase solution (KOD One PCR Master Mix, TOYOBO) and run for 40 cycles. After purification using the AMPure XP Purification Kit (Beckman Coulter), a second PCR was performed using the Nextera XT Index Kit (Illumina) and run for 8 cycles. The second PCR products were purified using the AMPure XP purification Kit, and libraries were pooled at equimolar concentrations. Sequencing was performed on an Illumina MiSeq platform using a MiSeq Reagent Kit v3 with paired-end reads (2 × 300 bp).

The combined copy number of 16S and 18S genes amplified using the 515Y and 926R primers in bulk, cylindrical pellet, ellipsoidal pellet, and other pellet samples were quantified using a SYBR Green-based qPCR assay. qPCR standards were prepared by cloning the synthetic sequence ECC5001 described by Tourlousse et al. [33]. qPCR reactions were conducted in 20-μL volumes containing 10 μL of polymerase solution (Thermo Scientific SYBR Green qPCR Master Mix, Thermo Fisher Scientific), 1 μL each of 10 μM forward and reverse primers, 2 μL of template DNA, and 6.0 μL of DNase/RNase-free water (Qiagen). Thermal cycling conditions consisted of an initial denaturation at 95°C for 1 min, followed by 40 cycles of denaturation at 95°C for 15 s and annealing/extension at 60°C for 60 s. A melting curve analysis was subsequently performed to check for primer-dimer amplification by increasing the temperature from 60°C to 95°C at a rate of 0.3°C s^−1^ following denaturation at 95°C for 60 s. The *r*^2^ values of the standard curves ranged from 0.996 to 0.998.

### Bioinformatics

Sequences obtained from amplicon sequencing were taxonomically identified using the QIIME 2 platform [34]. Sequences were processed using the pipeline described by McNichol et al. [32], whereby reads were first separated into those derived from 16S and 18S rRNA genes and subsequently denoised and screened for chimeras using the DADA2 plugin [35]. Rarefaction curves for 16S and 18S rRNA genes were generated using the R package *vegan*, confirming that amplicon sequence variant (ASV) richness reached saturation in each sample. ASVs derived from 16S and 18S rRNA genes were taxonomically classified using a naïve Bayesian classifier by comparison with the SILVA 138 [36] and PR2 version 5.00 [37] reference databases, respectively. ASVs classified as eukaryotic chloroplast 16S were further taxonomically resolved by comparison with the PR2 database version 5.00 [37]*. Synechococcus* exhibits substantial genetic diversity and is divided into multiple clades [38]. Each clade is characterized by distinct physiological and ecological traits [38], and recent studies commonly describe *Synechococcus* at the clade level [12, 39]. Accordingly, ASVs identified as *Synechococcus* and *Cyanobium* were subjected to BLAST searches with an e-value threshold of 1 × 10^−5^ against the Cyanorak database [40] and assigned to the clade corresponding to the top hit, with ≥98.3% similarity (6 bp difference) and 100% alignment coverage, following previous studies [32, 41]. In this region, *Synechococcus* clade II or III could not be distinguished because the top BLAST hit matched sequences from multiple clades with equal similarity. In this case, the possible clades were reported using “or” (e.g. clade II or III).

The DNA sinking flux of particle type z and period *j* (*F_z,j_*, copies m^−2^ d^−1^) was calculated using the qPCR-based copy number (*Q_z,j_*, copies), the carbon content of the DNA-extracted sample (E*_z,j_*, mgC), and the carbon flux of particle type z (*C_z,j_*, mgC m^−2^ d^−1^) as follows:


$$ {F}_{z,j}={Q}_{z,j}\times \frac{C_{z,j}}{E_{z,j}} $$

The carbon flux of each particle was obtained in subsections Sampling and Environmental Data Collection and Fecal Pellets  Subsampling. Here, for non-fecal particle samples, some non-fecal particles were inevitably removed together with fecal pellets during the separation process. Thus, the DNA sinking flux of non-fecal particle samples, *F_non-fecal particles_*, was estimated indirectly by subtracting the mean DNA sinking flux of each fecal pellet type from that of bulk samples as follows:


\begin{align*} {F}_{non- fecal\ particles,j}=&\,\,{F}_{bulk,j}-{F}_{cylindrical\ pellets,j}-{F}_{ellipsoidal\ pellets,j}\nonumber\\ &-{F}_{other\ pellets,j} \end{align*}

Next, the DNA sinking flux of taxon *i* in particle type *z* and period *j*, *F_z,j,i_*, was calculated using *F_z,j_* and amplicon sequencing results as follows:


$$ {F}_{z,\kern0.5em j,i}={F}_{z,j}\times \frac{R_{z,j,i}}{16{S}_{z,j}+18{S}_{z,j}\times{B}_{z,j}\times 1.33} $$

*16S_z,j_* and *18S_z,j_* denote the total numbers of 16S and 18S rRNA reads in particle type *z* and period *j*, and the number of reads assigned to taxon *i* in the amplicon sequencing is *R_z,j,i_*. Two known correction factors were applied: one is associated with Illumina sequencing and differences in qPCR fluorescence intensity arising from amplicon length differences between 16S and 18S rRNA genes [32], and the other one is based on the length ratio of the 16S and 18S amplicons [42]. The former (*B_z,j_*) was calculated in each sample based on McNichol et al. [32], and the latter was a constant at 1.33 [42].

### Data analysis

As an index of cyanobacterial contribution to carbon flux, the number of cyanobacterial 16S rRNA gene copies per unit carbon was calculated for each particle type. Comparisons among particle types and sampling periods were performed using one-way ANOVA followed by Welch’s *t-*test with false discovery rate (FDR) correction.

To identify organisms potentially regulating cyanobacterial sinking flux, Pearson correlation coefficients were calculated between fluxes of cyanobacterial 16S rRNA genes and class-level eukaryotic 18S rRNA genes. *P*-values were corrected for multiple comparisons using FDR. Least absolute shrinkage and selection operator (Lasso) regression [43] was also performed using cyanobacterial 16S rRNA gene flux as the response variable and class-level eukaryotic 18S rRNA gene fluxes as explanatory variables. Flux data were z-score standardized and subsequently analyzed using LassoCV implemented in the *scikit-learn* library in Python. Additionally, correlations among cyanobacterial 16S rRNA gene fluxes, carbon flux, and environmental variables were examined.

### Gel-trap observation

To collect sinking particles while preserving their original morphology and to examine cyanobacterial sinking forms in detail, drifting sediment traps containing RNA-fixative gels were deployed at a depth of 200 m at the same station for 24 h, from 24 to 25 July 2025. Collected particles were individually retrieved onboard and stored at −20°C. Particle morphology and cyanobacterial phycoerythrin fluorescence were observed using a scanning electron microscope (S-4800, Hitachi) and a fluorescence microscope (Axioskop 2 plus, Zeiss) with 579 nm excitation light. Detailed methodological descriptions are provided in the Supplementary Material.

## Results

### Surface environmental conditions

**Figure 2:**
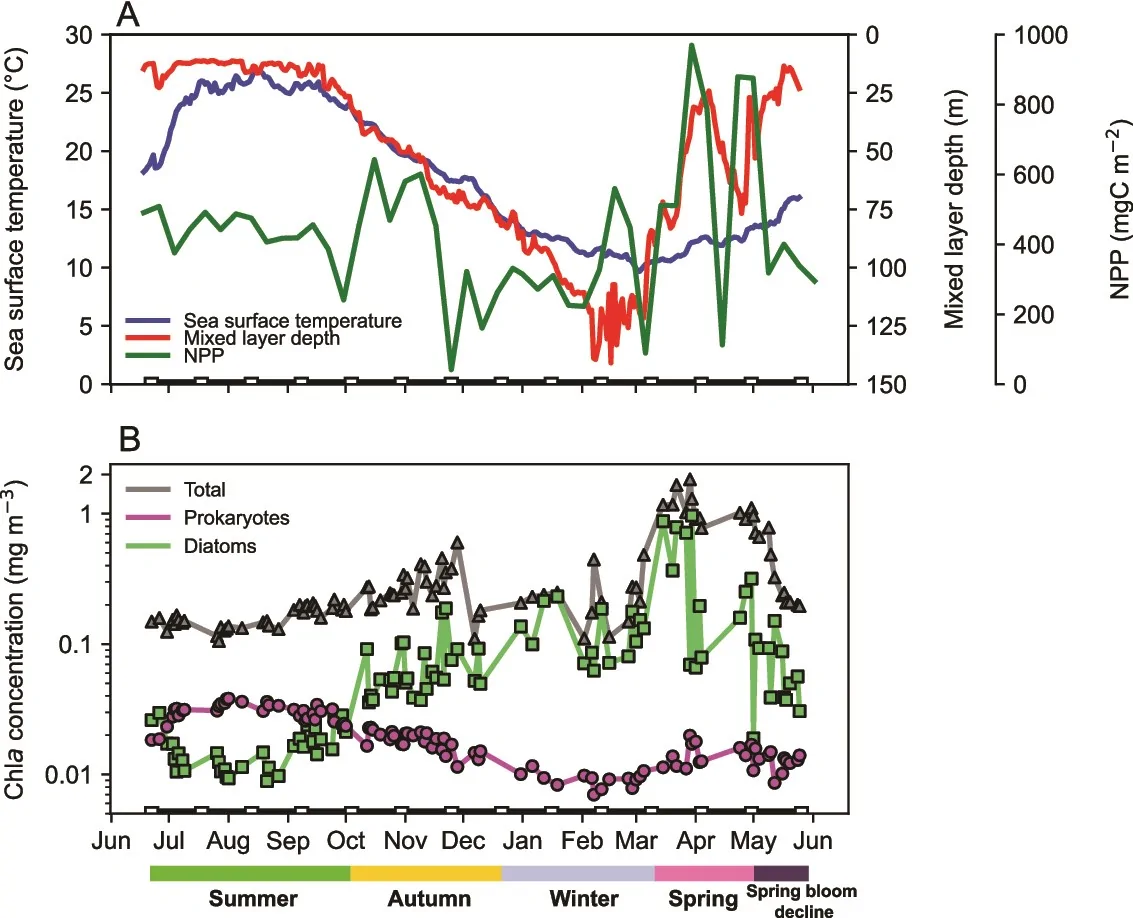
(A) Surface water temperature, mixed layer depth, NPP, and (B) chlorophyll *a* concentration derived from satellite-based data for total phytoplankton, prokaryotes, and diatoms from June 2022 to May 2023 at Stn. FATO (38° 43.2′ N, 137° 48′ E). The line along the *x*-axis represents the sediment trap deployment period, and white dots on the *x*-axis indicate the timing of receiving-cup switches. The bar at the bottom of the graph indicates the duration of each season. The temperature and mixed layer depth were from the MOVE/MRI.COM-JPN Dataset, and the others were from ocean-color satellite data.

Based on sea surface temperature, mixed layer depth, satellite-derived chlorophyll *a* concentration, and NPP, the sampling periods were categorized into five seasons (Fig. 2A and B): summer (June–September), characterized by water-column stratification and cyanobacterial dominance; autumn (October–December), during which the mixed layer began to deepen and the autumn bloom occurred; winter (January–early March), when sea surface temperature was lowest and the mixed layer deepest; spring (late March–April), when shoaling of the mixed layer triggered a diatom spring bloom; and the spring bloom decline phase (May), when NPP and chlorophyll *a* concentration decrease sharply. This seasonal variation in the phytoplankton community was also supported by the 16S rRNA gene amplicon sequencing data (Fig. S2).

### Components of sinking particles

**Figure 3:**
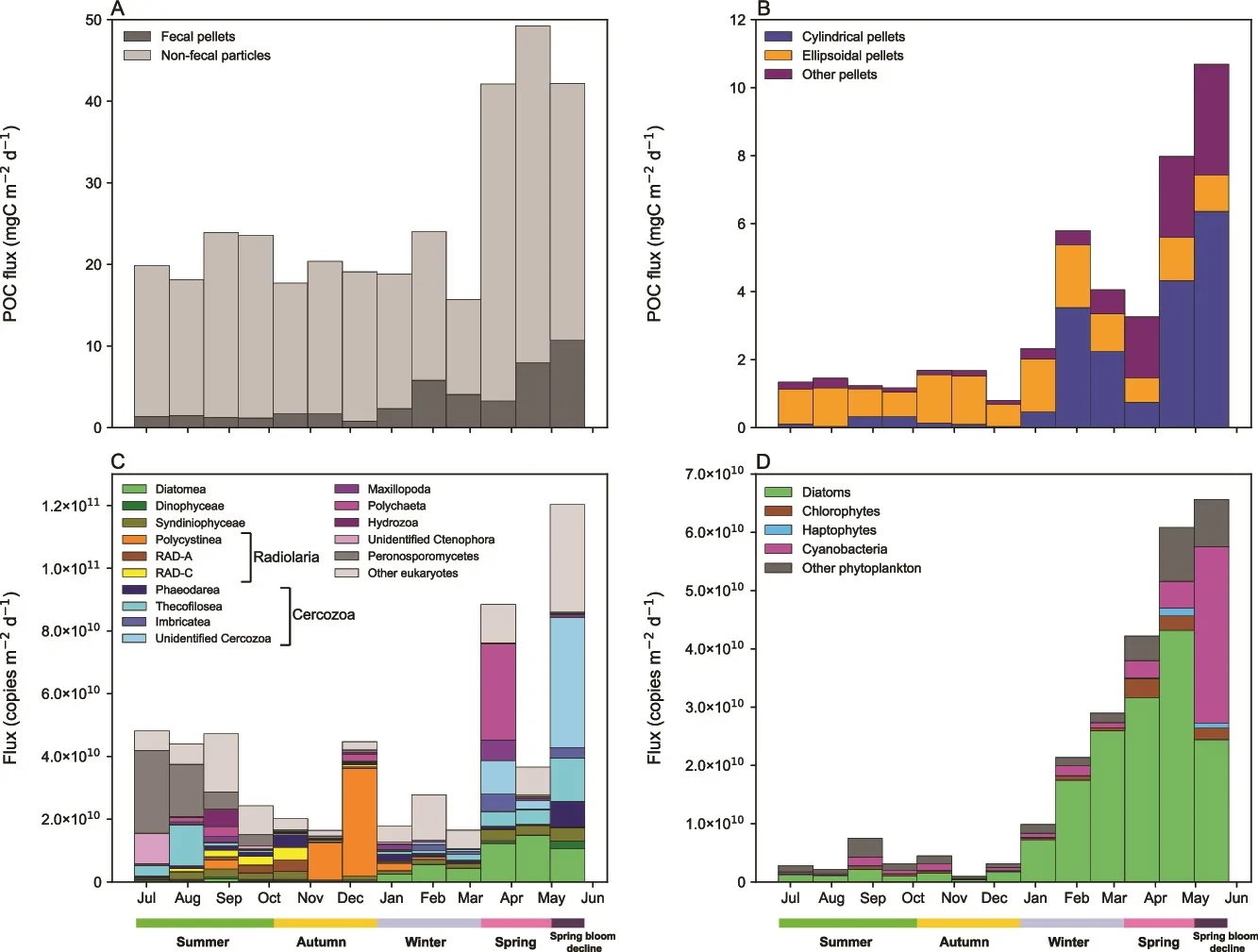
(A) Total POC flux, (B) breakdown of fecal pellet carbon flux, (C) eukaryotic flux at the class level based on 18S rRNA gene data, and (D) phytoplankton flux based on 16S rRNA gene data in sinking particles from June 2022 to May 2023. Panels (C) and (D) were derived from bulk samples and calculated using the carbon content of the DNA-extracted subsamples from the bulk sample, 18S (C) or 16S (D) rRNA gene copy numbers obtained by qPCR analysis, and relative abundance based on the amplicon sequencing. Bars at the bottom of panels (C) and (D) indicate the duration of each season.

Total POC flux increased to 42.1–49.3 mgC m^−2^ d^−1^ during spring, coincident with the surface phytoplankton bloom, whereas it remained relatively stable during other seasons at 15.7–20.4 mgC m^−2^ d^−1^ (Fig. 3). The contribution of fecal pellets to total POC flux ranged from 4.1% to 25.4%, with non-fecal particles remaining dominant throughout the year (Fig. 3A). Within fecal pellets, ellipsoidal pellets dominated from July to January, accounting for 61.2%–85.5% of fecal pellet carbon flux (Fig. 3B). Their flux ranged from 0.66 to 1.85 mgC m^−2^ d^−1^, indicating relatively consistent contributions throughout the year. In contrast, cylindrical pellets became dominant from winter through the spring bloom decline phase, accounting for 22.3%–60.9% of fecal pellet carbon flux. Their flux ranged from 0.03 to 6.36 mgC m^−2^ d^−1^, exhibiting substantial seasonal variability. From summer to autumn, Peronosporomycetes and radiolarians, including Polycystinea, RAD-A, and RAD-C, dominated eukaryotic assemblages in sinking particles based on 18S rRNA gene data (Fig. 3C). Among phytoplankton, Diatomea (diatoms) were the most abundant group; however, their contribution and sinking flux were relatively low, at 0.34%–2.05% and 5.56 × 10^7^–9.73 × 10^8^ copies m^−2^ d^−1^, respectively. From winter to spring, the contribution of Cercozoa—including Phaeodaria, Thecofilosea, Imbricatea, and unidentified Cercozoa—increased, and diatom contribution and sinking flux also increased, reaching 8.81%–40.2% and 2.44 × 10^9^ to 1.48 × 10^10^ copies m^−2^ d^−1^, respectively. Based on 16S rRNA gene data, diatoms accounted for 29.0%–89.7% of phytoplankton 16S rRNA reads and were the largest contributors; their sinking flux exhibited trends similar to those observed using 18S rRNA gene data (Fig. 3D). Cyanobacteria accounted for 3.11%–46.2% of phytoplankton reads, making them the second-largest contributors after diatoms based on the phytoplankton 16S rRNA reads. POC flux showed significant positive correlations with both surface-layer diatom biomass and diatom sinking flux (*P* < .05).

### Temporal variations of cyanobacteria sinking flux

**Figure 4:**
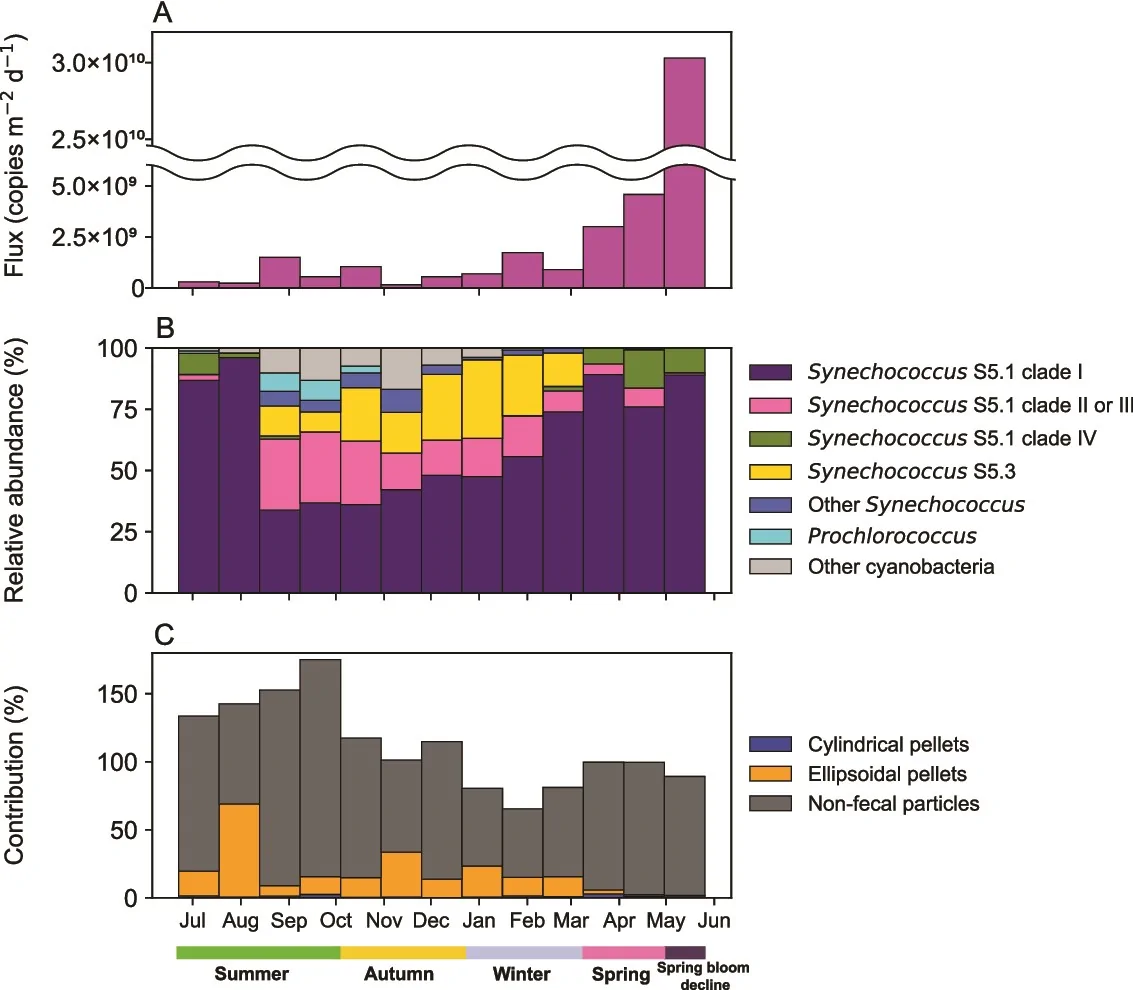
(A) Sinking flux of cyanobacteria, (B) cyanobacterial assemblage, and (C) contribution of each particle type to the cyanobacterial sinking flux based on 16S rRNA gene data in bulk sinking particles from June 2022 to May 2023. In (C), 100% indicates the cyanobacterial sinking flux of the bulk sample. The bar at the bottom of the graph indicates the duration of each season.

The sinking flux of cyanobacterial 16S rRNA genes increased to 3.01 × 10^9^ to 3.03 × 10^10^ copies m^−2^ d^−1^ in spring and showed values approximately one order of magnitude higher during the spring bloom decline phase; in contrast, no clear seasonality was observed during other seasons, with fluxes ranging from 1.59 × 10^8^ to 1.50 × 10^9^ copies m^−2^ d^−1^ (Fig. 4A). Among cyanobacteria, *Synechococcus* was the dominant group; in particular, *Synechococcus* subcluster 5.1 clade I accounted for 36.1%–96.1% of reads and predominated throughout the year (Fig. 4B). From summer to spring, subcluster 5.1 clade II or III (which could not be unambiguously distinguished) and subcluster 5.3 contributed 8.61%–29.0% and 8.19%–32.1%, respectively, indicating consistent contributions to cyanobacterial assemblages. Based on combined amplicon sequencing and qPCR analyses of bulk samples and non-fecal particles, 50.4%–159% of cyanobacterial sinking flux was attributed to the non-fecal particle fraction (Fig. 4C). Ellipsoidal pellets exhibited variable but persistent contributions, ranging from 0.38% to 68.6%, whereas cylindrical pellets contributed minimally, ranging from 0.08% to 2.96%. Values exceeding 100% reflect the variability of cyanobacterial contributions and uncertainty inherent to the indirect estimation approach, in which non-fecal particle flux was calculated as the difference between bulk and fecal pellet fluxes. The combined contributions of non-fecal particles, cylindrical pellets, and ellipsoidal pellets accounted for nearly equivalent to the bulk flux and occasionally exceeded it. Thus, the contribution of the other pellet fraction (mixture of spherical, damaged, and fragmented forms) was considered minor throughout the year, although it was not quantified separately.

### Efficiency of cyanobacteria transport

**Figure 5:**
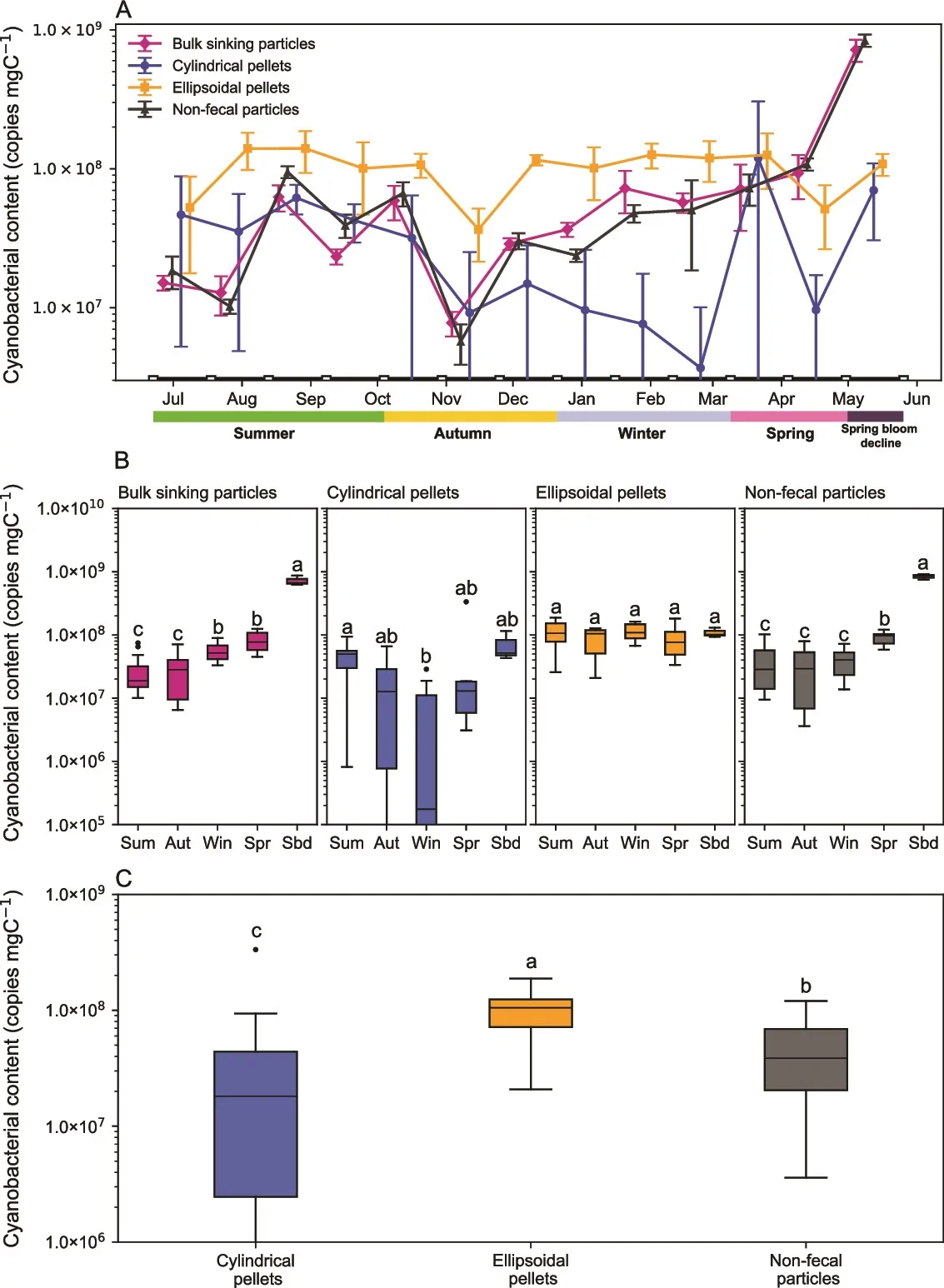
(A) Cyanobacterial 16S rRNA gene copy number per unit carbon in bulk sinking particles, cylindrical pellets, ellipsoidal pellets, and non-fecal particles from June 2022 to May 2023. Error bars represent the standard deviation. The bar at the bottom of the graph indicates the duration of each season. (B) Seasonal comparison of cyanobacterial 16S rRNA gene copy number per unit carbon for each particle type. The abbreviations sum, aut, win, spr, and sbd correspond to summer, autumn, winter, spring, and the spring bloom decline phase, respectively. (C) Comparison among particle types over the observation period, excluding the spring bloom decline phase. The *y*-axis of each panel is log-transformed.

Cyanobacterial 16S rRNA gene copy number per unit carbon differed among seasons and particle types (Fig. 5). In bulk sinking particles, cyanobacterial content in summer, when surface-layer biomass was highest (Figure 2B), was 2.84 × 10^7^ ± 2.19 × 10^7^ copies mgC^−1^ (mean ± SD), comparable to autumn values (3.19 × 10^7^ ± 2.38 × 10^7^ copies mgC^−1^) and significantly lower than values observed in winter (5.54 × 10^7^ ± 2.04 × 10^7^ copies mgC^−1^), spring (8.22 × 10^7^ ± 3.28 × 10^7^ copies mgC^−1^), and the spring bloom decline phase (7.18 × 10^8^ ± 1.30 × 10^8^ copies mgC^−1^) (*P* < .05) (Fig. 5B). During the spring bloom decline phase, values were approximately one order of magnitude higher than those observed during other seasons (*P* < .05). Non-fecal particles exhibited a trend similar to that observed in bulk sinking particles. In cylindrical pellets, cyanobacterial content was significantly higher in summer (4.66 × 10^7^ ± 2.56 × 10^7^ copies mgC^−1^) than in winter (6.99 × 10^6^ ± 1.05 × 10^7^ copies mgC^−1^) (*P* < .01). In contrast, no significant seasonal variation was observed in ellipsoidal pellets (*P* > .05). Excluding the spring bloom decline phase, cyanobacterial content was highest in ellipsoidal pellets (1.01 × 10^8^ ± 4.60 × 10^7^ copies mgC^−1^), followed by non-fecal particles (4.74 × 10^7^ ± 3.35 × 10^7^ copies mgC^−1^) and cylindrical pellets (3.27 × 10^7^ ± 5.74 × 10^7^ copies mgC^−1^) (*P* < .01) (Fig. 5C).

### Contribution of other organisms to cyanobacteria transport

**Figure 6:**
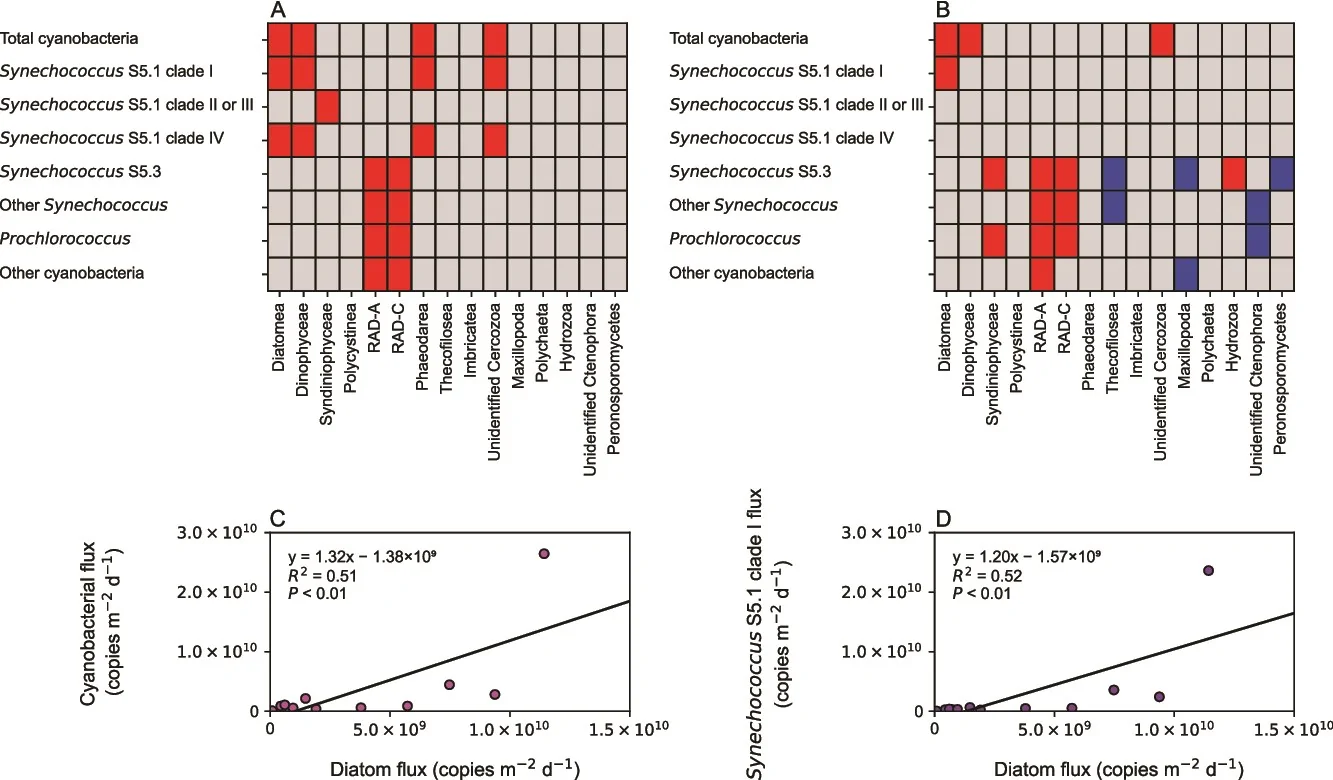
(A) Heatmap showing correlations cyanobacterial flux and eukaryotic flux in non-fecal particles. Significant positive correlations (*P* < .05) are shown in red, whereas nonsignificant correlations are shown in gray. (B) LASSO regression results for non-fecal particles, showing variables with positive coefficients in red, negative coefficients in blue, and variables not selected in gray. (C) Relationship between cyanobacterial flux and diatom flux and (D) relationship between *Synechococcus* subcluster 5.1 clade I flux and diatom flux in non-fecal particles. In (C) and (D), lines indicate linear regression fits; the equations and their statistical values (*R*^2^: the detection coefficient; *P*: *p*-value) were obtained from least-squares regression.

The variability in cyanobacterial sinking flux was primarily controlled by the amount sinking as non-fecal particles (Fig. 4C). In non-fecal particles, sinking fluxes of total cyanobacteria and of *Synechococcus* subcluster 5.1 clade I, which dominated the cyanobacterial community throughout the year, showed significant positive correlations with sinking fluxes of diatoms (Diatomea), dinoflagellates (Dinophyceae), Phaeodarea, and unidentified Cercozoa among the taxa shown in Fig. 3 (*P* < .05) (Fig. 6A, C, and  D). Furthermore, LASSO regression analysis identified diatoms as the only predictor variable for *Synechococcus* subcluster 5.1 clade I flux among the taxa shown in Fig. 3 (Fig. 6B). The positive relationships between the sinking fluxes of diatoms and cyanobacteria, and between diatoms and *Synechococcus* subcluster 5.1 clade I, remained significant (*P* < .01) even when excluding the decline phase of the spring bloom, a period of very high cyanobacterial sinking (Fig. S3). At the genus level, sinking fluxes of total cyanobacteria and *Synechococcus* subcluster 5.1 clade I showed significant positive correlations with *Chaetoceros*—the dominant diatom genus throughout the year—and with *Meuniera*, which contributed substantially to the spring bloom (*P* < .05) (Fig. S4). Carbon flux of non-fecal particles, as well as sea surface temperature and mixed layer depth, showed no significant correlations with cyanobacterial sinking flux (*P* > .05).

In cylindrical pellets, the sinking fluxes of total cyanobacteria and of *Synechococcus* subcluster 5.1 clade I showed significant positive correlations with the sinking fluxes of dinoflagellates, Thecofilosea, Imbricatea, and unidentified Cercozoa (*P* < .05), whereas no significant correlations with any taxa were observed in ellipsoidal pellets (Fig. S5).

## Discussion

Our study shows that a large proportion of cyanobacteria, primarily *Synechococcus*, was transported within non-fecal particles rather than fecal pellets. In non-fecal particles, the sinking flux of cyanobacteria was high during the spring diatom bloom (Fig. 4A), and a significant positive correlation was observed between the sinking flux of cyanobacteria and that of diatoms (Fig. 6A). These results suggest that the incorporation of cyanobacteria into diatom-containing aggregates during the spring bloom represents a major pathway by which they contribute to the BCP. Phycoerythrin fluorescence from cyanobacteria was observed within large diatom-containing aggregates collected using gel traps (Fig. S6), and the high sinking efficiency of *Synechococcus* during spring bloom has also been reported [44]. Diatoms release transparent exopolymer particles (TEP) [45], which increase particle stickiness [46] and promote aggregation with surrounding particles, including *Synechococcus*. Furthermore, the maximum sinking flux of *Synechococcus* was observed during the decline phase of the diatom bloom, which could be associated with increases in TEP production of diatoms and particle stickiness [47, 48].

Moreover, *Synechococcus* subcluster 5.1 clade I dominated the sinking cyanobacterial community. This clade is known to dominate in the colder water of the western North Pacific at latitudes higher than 35° N [49], and the actual observations supported the dominance of this clade in the Sea of Japan throughout the year (Fig. S7). This clade is more abundant in aggregate (>3 μm) than free-living (<3 μm) fractions [39], suggesting a higher propensity for incorporation into aggregates. This observation further supports the formation of aggregates with diatoms in the spring bloom. Although *Synechococcus* can form large aggregates under experimental conditions [17], such aggregation in natural environments appears limited to conditions involving mineral kaolinite ballast and high cell abundance [50]. In addition, the mismatch between surface cyanobacterial biomass and their contribution to POC flux indicates that cyanobacteria primarily sink through aggregation with other plankton, particularly diatoms, rather than through self-aggregation.

In contrast to diatoms, other eukaryotic plankton groups may exhibit secondary or indirect contributions to cyanobacterial export (Fig. 6). The positive relationship between phaeodarians and cyanobacteria suggests that phaeodarians contribute to cyanobacterial export within the mesopelagic layer rather than that occurring from the surface, given their mesopelagic habitat [51–54]. Phaeodarians possess gelatinous bodies and siliceous skeletons that enable particle binding and the formation of ballasted aggregates [55]. Other cercozoans, excluding phaeodarians, are largely considered parasitic; few species have been described, and their ecological roles remain poorly understood [52, 54]. Therefore, the mechanisms by which unidentified cercozoans contribute to cyanobacterial sinking remain unknown. The sinking flux of dinoflagellates also showed a positive correlation with cyanobacterial sinking flux (Fig. 6A). Their contribution to sinking particles was low (Fig. 3C), suggesting that dinoflagellates do not play a major role in cyanobacterial sinking; however, they may contribute indirectly by grazing on cyanobacteria and subsequently sinking themselves [11] or by promoting aggregation through the production of mucospheres [56].

Cyanobacterial transport via fecal pellets represents not a major pathway in our study, although ellipsoidal pellets exhibited high transport efficiency (Fig. 5C) and made a considerable contribution during periods outside of the diatom spring bloom (Fig. 4C). On the other hand, the contribution of cylindrical pellets was mostly negligible (Fig. 4C). This difference is thought to reflect differences in the feeding mode of the fecal pellet producers. Krill and copepods are unable or unlikely to feed directly on pico- and nano-sized prey [57], while appendicularians capture particles using mucous nets, enabling them to feed on these small-sized prey [58]. Thus, the presence of cyanobacteria in ellipsoidal fecal pellets is expected. Moreover, ellipsoidal pellets observed at this depth at this station have been reported to originate from mesopelagic appendicularians [14]. These mesopelagic appendicularians inhabit not only the Sea of Japan but also a wide area of the western North Pacific [20]. Therefore, zooplankton, particularly mesopelagic appendicularians, may play an important role in cyanobacterial sinking during periods of low diatom biomass. The sinking fluxes of cyanobacteria in ellipsoidal pellets showed no positive correlations with any taxa (Fig. S5). This may reflect biases in the taxonomic composition of fecal pellets caused by digestive processes and feeding habitats.

Several caveats should be noted in this study. It was not possible to directly quantify the contribution of cyanobacteria to POC flux or to assess the impact of variations in their sinking efficiency on the BCP. For example, although copy number of *Synechococcus* or phycoerythrin fluorescence has been converted to carbon content to estimate contributions to POC flux in previous studies [12, 59], such a conversion was not feasible here due to low DNA extraction efficiency and fluorescence loss caused by formalin fixation [60]. It should also be noted that the copy numbers of 16S and 18S rRNA genes per unit of carbon vary among taxonomic groups [61, 62], and therefore, the results of this study do not necessarily reflect the exact contributions to carbon flux. In addition, the quantitative evaluation of the relationship between diatoms and cyanobacteria in this study is based solely on DNA data and does not directly elucidate the underlying mechanisms operating in the surface ocean. Future studies employing experimental approaches are required to elucidate the mechanisms governing aggregate formation between diatoms and cyanobacteria.

In conclusion, our study demonstrates that (i) cyanobacteria are transported to the deep ocean in a non-negligible amount and that (ii) their sinking occurs with diatom sinking, especially in the spring bloom. These findings illustrate how interactions among phytoplankton groups contribute to the functioning of the BCP. The presence of large, ballasted phytoplankton strengthens the BCP not only through their own sinking but also by facilitating the sinking of smaller phytoplankton. However, the magnitude of this process is likely regulated by surface environmental conditions. Therefore, to assess the spatiotemporal efficiency of carbon sequestration, future studies should aim to elucidate how these interactions are regulated.

## Supplementary Material

Supplementary_material_ycag183

## Data Availability

All sequence reads are available in the DDBJ Bioproject database under accession number PRJDB37391. All other data are available on Figshare (10.6084/m9.figshare.30762515).
