# Supplementary material for: Enhanced Synechococcus export associated with diatom sinking
Source: ISME Commun. 2026 Jul 14;6(1):ycag183. doi: 10.1093/ismeco/ycag183 (PMC13503964; doi:10.1093/ismeco/ycag183)
Supplement: Supplementary file 1 [file supplementary_material_ycag183.zip › Supporting_ISMECOMMUN-D-25-00784R2_ycag183.docx]

**Supplementary material for**

Enhanced *Synechococcus* Export Associated with Diatom Sinking

Takehiro Shimonaka^1^, Taketoshi Kodama^1^, Shigeyoshi Otosaka^2^, Taku Wagawa^3^, Takafumi Kataoka⁴, Junya Hirai^2^, Kazutaka Takahashi^1^

^1^Graduate School of Agricultural and Life Sciences, The University of Tokyo, Tokyo, 113-8657, Japan

^2^Atmosphere and Ocean Research Institute, The University of Tokyo, Kashiwa, 277-8654, Japan

^3^Fisheries Resources Institute, Japan Fisheries Research and Education Agency, Niigata, 951-8121, Japan

^4^Faculty of Marine Science and Technology, Fukui Prefectural University, Obama, 917-0003, Japan

Corresponding author: Taketoshi Kodama (takekodama@g.ecc.u-tokyo.ac.jp)

**Contents of this file**

Supplementary method for gel trap observation

Figures S1 to S7

Supplementary references

**Supplementary method for gel trap observation**

The drifting sediment traps were deployed at a depth of 200 m at Stn. FATO for 24 hours, from July 24 to July 25, 2025. The trap tubes contained 0.2 μm-filtered seawater overlying RNA-fixative gel layers. The RNA-fixative gel layers were prepared as described in Baumas et al. [1]. The recovered traps were left to stand for one hour, after which the water above was removed using a siphon [2]. The collected particles were individually picked up with a p1000 pipettor under a stereo microscope, transferred into cryovials, and stored at –20 °C. After being thawed in the laboratory, the particles were transferred into Milli-Q water to remove the RNA-fixative gel. For the scanning electron microscopy samples, they were sequentially dehydrated in 50% and then 99.5% ethanol, and subsequently sputter-coated with platinum-palladium. Particles and phycoerythrin fluorescence of cyanobacteria were observed using a scanning electron microscope (S-4800, Hitachi) and a fluorescence microscope (Axioskop 2 plus, Zeiss) with 579 nm excitation light.

**Fig. S1. Representative fecal pellets in the sediment trap samples: (A) cylindrical (considered to originate from large copepods or krill), (B) ellipsoidal (considered to originate from appendicularians), (C) others (other shapes), and (D) others (fragmented). The scale bar represents 200 μm.**

**Fig. S2 Phytoplankton assemblages based on 16S rRNA gene read numbers in suspended particles at 50 m depth: (A) from June 2016 to June 2017 at St3, 7, 9, and 13, and (B) in June 2022 at Station SI05.**

**Fig. S3 (A) Relationship between cyanobacterial flux and diatom flux, and (B) relationship between *Synechococcus* subcluster 5.1 clade I flux and diatom flux in non-fecal particles, excluding thedelcine phase of the spring bloom. Lines in each panel indicate linear regression fits.**

**Fig. S4 (A) Diatom assemblages at the genus level in the non-fecal particles based on the 18S rRNA gene and (B)** **heatmap showing correlations between cyanobacterial flux in the non-fecal particles and diatom flux at the genus level. The p values were FDR-adjusted, with significant positive correlations (*P* < 0.05) shown in red, and non-significant correlations displayed in gray.**

**Fig. S5. Heatmap showing correlations cyanobacterial flux and eukaryotic flux in (A) cylindrical pellets and (B) ellipsoidal pellets.** **Significant positive correlations (*P* < 0.05) are shown in red, whereas non-significant correlations are shown in gray.**

**Fig. S6** **Particles collected by the gel trap were observed under (A) a light microscope, (B) a scanning electron microscope, where diatom frustules are visible, and (C) a fluorescence microscope, where** **the phycoerythrin fluorescence signals of cyanobacteria are visible in yellow. The scale bar in (A) is 200 µm, and the scale bars in (B) and (C) are 20 µm. The particle shown in (A) and (C) is the same.**

**Fig. S7** **Cyanobacterial assemblages based on 16S rRNA gene read numbers in suspended particles at 50 m depth: (A) from June 2016 to June 2017 at St3, 7, 9, and 13, and (B) in June 2022 at Station SI05.**

Supplementary references

1.	Baumas CMJ, Ababou F-E, Garel M *et al.* A novel method to sample individual marine snow particles for downstream molecular analyses. *Limnol Oceanogr Methods* 2024;**22**:34-46. <https://doi.org/10.1002/lom3.10590>

2.	Durkin CA, Cetinić I, Estapa M *et al.* Tracing the path of carbon export in the ocean though DNA sequencing of individual sinking particles. *ISME J* 2022;**16**:1896-906. <https://doi.org/10.1038/s41396-022-01239-2>
